# Pharmacological inhibition of Akt and downstream pathways modulates the expression of COX-2 and mPGES-1 in activated microglia

**DOI:** 10.1186/1742-2094-9-2

**Published:** 2012-01-03

**Authors:** Antonio CP de Oliveira, Eduardo Candelario-Jalil, Julia Langbein, Lena Wendeburg, Harsharan S Bhatia, Johannes CM Schlachetzki, Knut Biber, Bernd L Fiebich

**Affiliations:** 1 Department of Psychiatry and Psychotherapy, University of Freiburg Medical School, Hauptstr. 5, D-79104 Freiburg, Germany; 2 Department of Pharmacology, Universidade Federal de Minas Gerais, Av. Antonio Carlos 6627, 31270-901, Belo Horizonte, Brazil; 3 Department of Neuroscience, University of Florida, Gainesville, FL 32610, USA; 4 Department of Molecular Neurology, University of Erlangen, Erlangen, Germany; 5 VivaCell Biotechnology GmbH, Ferdinand-Porsche-Str. 5, D-79211, Denzlingen, Germany

**Keywords:** microglia, phosphatidylinositol 3-kinase, mammalian target of rapamycin, glycogen synthase kinase-3, Akt, prostaglandins

## Abstract

**Background:**

Microglia are considered a major target for modulating neuroinflammatory and neurodegenerative disease processes. Upon activation, microglia secrete inflammatory mediators that contribute to the resolution or to further enhancement of damage in the central nervous system (CNS). Therefore, it is important to study the intracellular pathways that are involved in the expression of the inflammatory mediators. Particularly, the role of the phosphatidylinositol 3-kinase (PI3K)/Akt/mammalian target of rapamycin (mTOR) and glycogen synthase kinase-3 (GSK-3) pathways in activated microglia is unclear. Thus, in the present study we investigated the role of Akt and its downstream pathways, GSK-3 and mTOR, in lipopolysaccharide (LPS)-activated primary rat microglia by pharmacological inhibition of these pathways in regard to the expression of cyclooxygenase (COX)-2 and microsomal prostaglandin E synthase-1 (mPGES-1) and to the production of prostaglandin (PG) E_2 _and PGD_2_.

**Findings:**

We show that inhibition of Akt by the Akt inhibitor X enhanced the production of PGE_2 _and PGD_2 _without affecting the expression of COX-2, mPGES-1, mPGES-2 and cytosolic prostaglandin E synthase (cPGES). Moreover, inhibition of GSK-3 reduced the expression of both COX-2 and mPGES-1. In contrast, the mTOR inhibitor rapamycin enhanced both COX-2 and mPGES-1 immunoreactivity and the release of PGE_2 _and PGD_2_. Interestingly, NVP-BEZ235, a dual PI3K/mTOR inhibitor, enhanced COX-2 and reduced mPGES-1 immunoreactivity, albeit PGE_2 _and PGD_2 _levels were enhanced in LPS-stimulated microglia. However, this compound also increased PGE_2 _in non-stimulated microglia.

**Conclusion:**

Taken together, we demonstrate that blockade of mTOR and/or PI3K/Akt enhances prostanoid production and that PI3K/Akt, GSK-3 and mTOR differently regulate the expression of mPGES-1 and COX-2 in activated primary microglia. Therefore, these pathways are potential targets for the development of novel strategies to modulate neuroinflammation.

## Findings

Inflammation has been recognized not only as a mere bystander in neurodegenerative diseases but also as a factor driving disease progression. Microglia, the innate phagocytic cells of the central nervous system (CNS), constantly survey their microenvironment. Activated microglia secrete inflammatory mediators, which could contribute to neuronal damage. Different groups have demonstrated that the inflammatory cyclooxygenase-2 (COX-2), inducible nitric oxide synthase (iNOS) and cytokines, such as interleukin (IL)-1β, IL-6 and tumor necrosis factor (TNF)-α, are associated with neurodegenerative diseases [1]. Thus, reduction of microglia activation is an important target in the treatment of neurodegenerative diseases. Therefore, much effort has been made to identify intracellular pathways that are responsible for the expression of these pro-inflammatory mediators. However, many intracellular pathways which are involved in the production of inflammatory mediators by microglia are not well characterized. In particular, the role of the phosphatidylinositol 3-kinase (PI3K) signal cascade in mediating neuroinflammatory processes is poorly studied.

The PI3K pathway can be activated by different stimuli including LPS via the toll-like receptor 4/CD14 receptor complex in microglia. After activation, PI3K phosphorylates phosphatidylinositol 4,5-bisphosphate to generate phosphatidylinositol-3,4,5-trisphosphate. The latter molecule binds to the pleckstrin homology domain of one of the Akt (also known as protein kinase B) isoforms and facilitates the phosphorylation of Akt1, Akt2 or Akt3 at Thr^308/309/305 ^and Ser^273/474/472^, respectively, by the phosphatidylinositol-dependent kinases 1 and 2 [2]. The phosphorylation on the respective residues of Akt leads to further catalytic activity changes of downstream targets, such as glycogen synthase kinase-3 (GSK-3) and mammalian target of rapamycin (mTOR) [3,4].

Recently, we and others have demonstrated that PI3K might play an important role in inflammation and microglia activation. In particular, we have demonstrated that COX-2 is up-regulated and microsomal prostaglandin E synthase-1 (mPGES-1) is down-regulated by the PI3K inhibitor LY294002 [5]. However, downstream pathways of PI3K might also be important. In order to investigate this issue, we utilized a pharmacological approach to further investigate the role of PI3K and downstream pathways in the expression of COX-2 and mPGES-1 by activated microglia.

Primary microglial cell cultures were established from cerebral cortices of one-day neonatal Wistar rats [6] as described in detail in our recent study [5]. The purity of the microglial culture obtained in our experiments was > 98% as determined by immunofluorescence and cytochemical analysis according to the method developed by Gebicke-Haerter et al. (1989) [7]. To investigate the effect of the inhibition of downstream pathways of PI3K, the following compounds were used: the PI3K inhibitors LY294002 and PI828, as well as LY303511, the inactive analogue of LY294002 (all from Tocris, Ellisville, MO, or Calbiochem, Bad Soden, Germany); Akt inhibitor X and mTOR inhibitor rapamycin (both from Calbiochem, Bad Soden, Germany); the dual PI3K/mTOR inhibitor NVP-BEZ235 (Axon Medchem BV, Groningen, The Netherlands); the GSK-3 inhibitor SB216763 (Tocris, Ellisville, MO); LPS (from *Salmonella typhimurium*, Sigma-Aldrich, Taufkirchen, Germany). Stock solutions (5-10 mM) were prepared in dimethyl sulfoxide (DMSO) and stored at -20°C. Further dilutions were carried out in DMSO and final concentration of DMSO for all concentrations of the drugs in culture medium was 0.1%. All compounds, used at the given concentrations, did not affect the viability of the cells as observed through the MTT cell viability assay (data not shown).

To analyze COX-2 and mPGES-1 protein levels, cells were incubated with the respective inhibitors for 30 min followed by 48 h stimulation with LPS. In the analysis of phosphorylation of p-70S6K, a downstream target of mTOR, cells were incubated with the inhibitors for 30 min followed by 1 h stimulation with LPS. 30 to 50 μg of protein from each sample was subjected to SDS-PAGE on a 10-15% gel under reducing conditions. Primary antibodies were goat anti-COX-2 (M-19, Santa Cruz, Heidelberg, Germany) diluted 1:500 in Tris-buffered saline (TBS) containing 0.1% Tween 20 (Merck, Darmstadt, Germany) and 1% bovine serum albumin (BSA, Sigma-Aldrich), rabbit anti-mPGES-1 (Oxford Biomedical Research, 1:1000), rabbit anti-phospho-p70S6K (Cell Signaling Technology, Beverly, MA, USA, 1:1000), rabbit anti-actin (Sigma, 1:5000). Proteins were detected with horseradish peroxidase (HRP)-coupled rabbit anti-goat IgG (Santa Cruz, 1:100,000) or HRP-coupled donkey anti-rabbit (GE Healthcare, Freiburg Germany, 1:25,000) using chemiluminescence (ECL) reagents (GE Healthcare).

To investigate the effect of Akt inhibitor X on cytosolic prostaglandin E synthase (cPGES) and mPGES-2, we performed real time PCR. Cells were pre-incubated with Akt X inhibitor at different concentrations (0.1 - 5 μM) and LPS (10 ng/ml) was subsequently added for total 24 h. RNA preparation was done by using RNAspin mini RNA isolation kit (GE Healthcare) and for cDNA synthesis one microgram of total RNA was reverse transcribed using M-MLV reverse transcriptase and random hexamers (Promega, Mannheim, Germany). The synthesized cDNA was the template for the real-time polymerase chain reaction (PCR) amplification carried out by the CFX96 real-time PCR detection system (Bio-Rad Laboratories Inc.). Specific Probes and primers were designed by using Universal probe library (Roche). Reaction conditions were 5 min at 95°C, followed by 40 cycles of 10 s at 95°C, 30 s at 60°C, and 1 s at 72°C followed by 10 s at 40°C. S12 served as an internal control for sample normalization and the comparative cycle threshold Ct method was used for data quantification as described previously [8]. The following primer sequences were used for mPGES-2: Forward 5'-AGGAAGGTACCCATCCTGGT-3', Reverse 5'-GAGGAGTCATTGAGCTGTTGC-3'; cPGES: Forward 5'-TGTCTAATTTTGACCGTTTCTCTG-3', Reverse 5'-TCATCTGCTCCGTCTACTTCTG-3'; S12: Forward 5'-GCGCTTAAATACCGTCATGC-3', Reverse: 5'-GACGCCGAATCTTGAACG-3'.

To determine PGE_2 _and PGD_2 _concentrations, cells were incubated with the respective inhibitors previously for 30 min followed by 48 h stimulation with LPS. Supernatants were harvested for the measurement of the levels of PGD_2 _(Cayman Chemicals, Ann Arbor, MI, USA) and PGE_2 _(AssayDesign, distributed by Biotrend, Köln, Germany). All measurements were performed according to the manufacturer's instructions. The standards were used in the interval of 39-2500 pg/ml (sensitivity of the assay was 36.2 pg/ml) for both prostaglandins.

All experiments were carried out at least three times. Original data were converted into % - values of LPS control and mean ± S.E.M. were calculated. Values were compared using *t*-test (two groups) or one-way ANOVA with *post-hoc *Student-Newman-Keuls test (multiple comparisons). The level of statistical significance was set at a p value less than 0.05.

**Figure 1:**
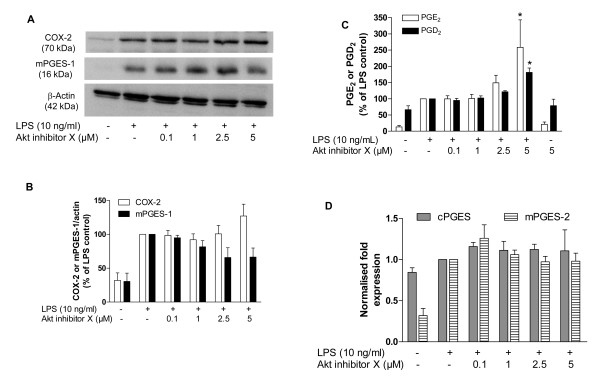
**Effect of Akt inhibitor X on COX-2 and mPGES-1 immunoreactivity and PGE_2 _and PGD_2 _release in primary LPS-stimulated rat microglia**. **A: **Immunoblot analysis of protein levels of COX-2, mPGES-1 and β-actin in LPS-activated microglia treated with Akt inhibitor X (0.1-5 μM). **B**: Quantitative densitometric analysis of COX-2 and mPGES-1 protein expression normalized to β-actin loading control. **C**: Effect of Akt inhibitor X (0.1-5 μM) on PGE_2 _and PGD_2 _production after 48 h of LPS stimulation in rat primary microglia. **D**: Effect of Akt inhibitor X on mPGES-2 and cPGES expression in primary LPS-stimulated rat microglia. *P < 0.05 with respect to LPS control.

We have recently demonstrated that LY294002, a PI3K inhibitor, reduces mPGES-1 and increases COX-2 expression, providing an interesting pattern of differential expression between these two enzymes [5]. To further investigate the contribution of pathways downstream of PI3K, we inhibited Akt with Akt inhibitor X. As shown in Figure 1, Akt inhibitor X slightly increased COX-2 and reduced mPGES-1 protein levels induced by LPS, although without reaching statistical difference (Figure 1A-B). On the other hand, the Akt inhibitor X increased significantly the production of PGE_2 _and PGD_2 _in LPS-activated microglia (Figure 1C). To investigate whether the enhancement of PGE_2 _was due to an increase in the expression of other prostaglandin synthases, we investigated the effect of Akt inhibitor X on mPGES-2 and cPGES. Interestingly, we found mPGES-2 to be increased after 24 h stimulation with LPS, albeit cPGES levels remained unchanged. However, Akt inhibitor X did not affect the expression of both enzymes (Figure 1D).

**Figure 2:**
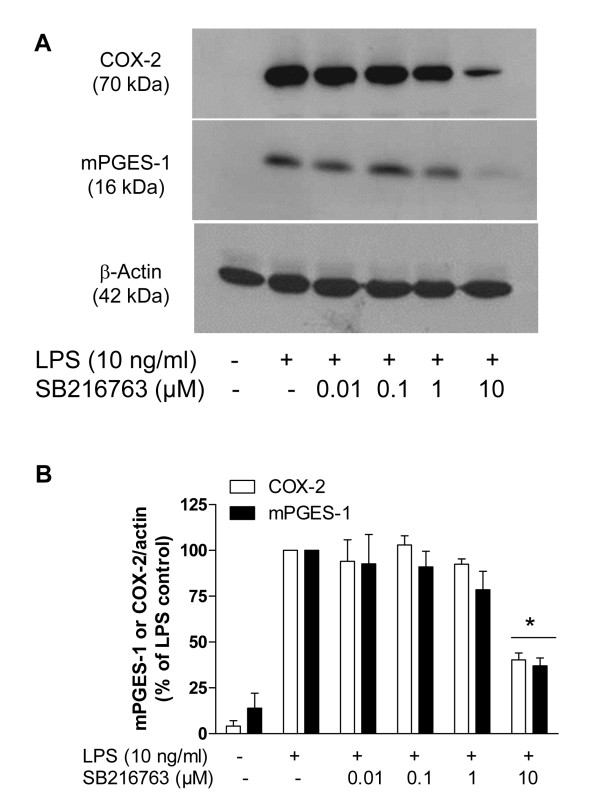
**Effect of SB216763, a GSK-3 inhibitor, on mPGES-1 and COX-2 immunoreactivity in primary LPS-stimulated rat microglia**. **A**: Immunoblot analysis of protein levels of COX-2, mPGES-1 and β-actin in LPS-activated microglia treated with SB216763 (0.01-10 μM). **B**: Quantitative densitometric analysis of mPGES-1 and COX-2 protein expression normalized to β-actin loading control. *P < 0.05 with respect to LPS control.

Since the involvement of Akt in the enhanced production of prostanoids was suggested by using Akt inhibitor X, we asked whether other downstream targets of PI3K/Akt are affected. An important target of Akt is GSK-3. Different studies have demonstrated that Akt inactivates GSK-3 by phosphorylating a serine residue of this enzyme [9]. This means that blockade of PI3K/Akt keeps GSK-3 in the active state, which might lead to increased COX-2 levels. Thus, inhibition of GSK-3 could potentially decrease COX-2 expression. Our data showed that the higher dose of the GSK-3 inhibitor SB216763 (10 μM), significantly decreased COX-2 and mPGES-1 immunoreactivity induced by LPS in primary rat microglia (Figure 2A-B).

**Figure 3:**
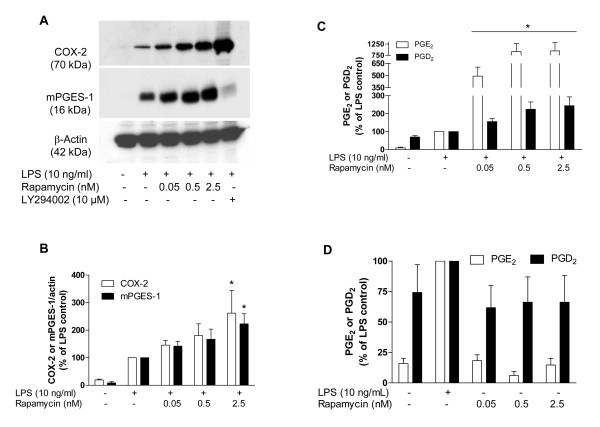
**Effect of rapamycin, a mTOR inhibitor, and NVP-BEZ235, a dual PI3K/mTOR inhibitor, on COX-2 and mPGES-1 immunoreactivity and PGE_2 _and PGD_2 _release in primary LPS-stimulated rat microglia**. **A: **Immunoblot analysis of protein levels of COX-2, mPGES-1 and β-actin in LPS-activated microglia treated with rapamycin (0.05-2.5 nM). **B**: Quantitative densitometric analysis of COX-2 and mPGES-1 protein expression normalized to β-actin loading control. **C**: Effect of rapamycin (0.05-2.5 nM) on PGE_2 _and PGD_2 _production after 48 h of LPS stimulation in rat primary microglia. **D**: Effect of incubation of rat microglia with rapamycin (0.05-2.5 nM) on PGE_2 _and PGD_2 _release in absence of LPS. *P < 0.05 with respect to LPS control.

Thereafter, we asked whether mTOR inhibition alters LPS-induced COX-2 and mPGES-1 protein synthesis. Interestingly, rapamycin, a well-known mTOR inhibitor, increased both COX-2 and mPGES-1 immunoreactivity (Figure 3A-B). The production of PGE_2 _and PGD_2 _was also strongly enhanced by rapamycin in LPS-stimulated microglia (Figure 3C), but not in non-stimulated cells (Figure 3D).

**Figure 4:**
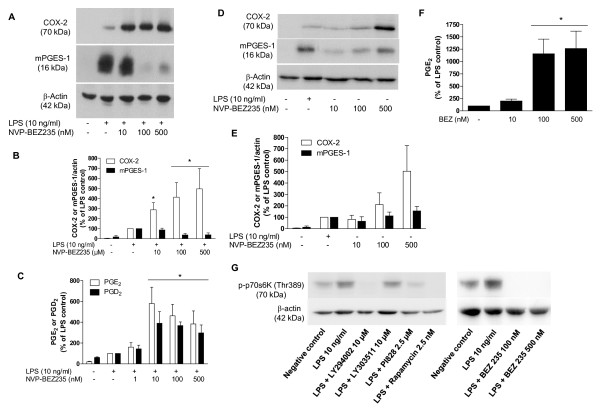
**Effect of NVP-BEZ235, a dual PI3K/mTOR inhibitor, on COX-2 and mPGES-1 immunoreactivity and PGE_2 _and PGD_2 _release in primary LPS-stimulated rat microglia**. **A: **Immunoblot analysis of protein levels of COX-2, mPGES-1 and β-actin in LPS-activated microglia treated with NVP-BEZ235 (10-500 nM). **B**: Quantitative densitometric analysis of COX-2 and mPGES-1 protein expression normalized to β-actin loading control. **C**: Effect of NVP-BEZ235 (10-500 nM) on PGE_2 _and PGD_2 _production after 48 h of LPS stimulation in rat primary microglia. **D**: Effect of incubation of rat microglia with NVP-BEZ235 (10-500 nM) on COX-2 and mPGES-1 in absence of LPS. **E**: Quantitative densitometric analysis of COX-2 and mPGES-1 protein expression normalized to β-actin loading control. For COX-2, there is a trend toward statistical significance (P = 0.0675). **F**: Effect of incubation of rat microglia with NVP-BEZ235 (10-500 nM) on PGE_2 _release in absence of LPS. **G**: Effect of PI3K inhibitors (LY294002 and PI828), LY303511 (inactive analogue of PI3K inhibitor), rapamycin (mTOR inhibitor) and NVP-BEZ235 (PI3K/mTOR dual inhibitor) on p70S6K phosphorylation. *P < 0.05 with respect to LPS control.

Considering the possible involvement of PI3K and mTOR in the regulation of mPGES-1 and COX-2 in microglia, we investigated the effect of NVP-BEZ235, a dual PI3K/mTOR inhibitor. NVP-BEZ235 strongly enhanced COX-2, but reduced mPGES-1 immunoreactivity in activated microglia (Figure 4A-B). The synthesis of PGE_2 _and PGD_2 _was also strongly enhanced (Figure 4C). We further investigated whether NVP-BEZ235 increases COX-2 and mPGES-1, as well as prostaglandin synthesis, in absence of LPS. As shown in Figure 4D-E, there was a trend to enhancement of COX-2 and mPGES-1 expression. However, PGE_2 _synthesis was increased in non-stimulated microglia (Figure 4F).

As expected, the PI3K inhibitors, LY294002 and PI828, as well as rapamycin and NVP-BEZ235, reduced the phosphorylation of p-70S6K, an indirect marker of mTOR activation (Figure 4G). The PI3K inactive analogue LY303511 did not change this parameter.

In the present study, we investigated the role of Akt and downstream pathways in microglia activation with special emphasis on the arachidonic acid cascade. We provide new evidence that inhibition of these pathways significantly influence microglia activation.

We have previously demonstrated that PI3K inhibition reduced mPGES-1 and increased COX-2 synthesis in activated microglia. Here we further investigated whether inhibition of Akt and downstream pathways contributes to the regulation of COX-2 and mPGES-1 in LPS-stimulated primary microglia. Although there was a trend towards an increased expression of COX-2 and a reduction in the expression of mPGES-1 with the exposure of the cells to Akt inhibitor X, no statistical difference was observed (Figure 1A-B). We have also observed that Akt inhibitor X did not change the expression of mPGES-2 and cPGES. However, as can be observed in Figure 1D, mPGES-2 is enhanced after 24 h incubation of cells with LPS. Although most studies indicate that mPGES-2 is a constitutive enzyme that is not inducible, the finding of the present study is interesting and is in accordance with some data that indicates that mPGES-2 can be enhanced in some conditions [10-12]. For example, Chaudhry et al. [10] have shown that mPGES-2 is expressed in activated, but not resting microglia of humans.

The highest dose of Akt inhibitor X significantly increased PGE_2 _and PGD_2_, indicating that a little enhancement in COX-2 expression is sufficient to increase prostanoid production (Figure 1C). The enhancement of PGE_2 _and PGD_2 _could be due to the slight, though not significant, enhancement of COX-2 expression. However, there are some other possibilities that could contribute to the enhancement of PGE_2 _and PGD_2_. Akt inhibition could enhance the expression and activity of PLA_2 _and COX-1, therefore enhancing the available arachidonic acid and PGH_2_, which are substrates for the synthesis of prostanoids. Moreover, it is possible that Akt inhibitor X could also enhance the enzymatic activity of the COX-2 and the PGE and PGD synthases.

We further investigated downstream pathways of Akt, such as GSK-3. As expected, a selective GSK-3 inhibitor reduced COX-2 and mPGES-1 immunoreactivity (Figure 2A-B). Although SB216763 has an IC_50 _in the nM range, it needs to be emphasized that this determination was performed with the isolated human enzyme [13], a condition that differs from the studies that use whole cells. In fact, different pharmacological effects have been demonstrated in macrophages and microglia exposed to this higher concentration [14-18].

Similar to our results, Takada et al. [19] have shown that genetic deletion of GSK-3β reduces the activation of NF-κB and COX-2 expression induced by TNFα-stimulated mouse embryonic fibroblasts. On the other hand, previous work has shown that GSK-3β inhibition results in COX-2 expression [20-22]. Moreover, Yuskaitis and Jope [18] showed that GSK-3 inhibition by the use of different GSK-3 inhibitors is not important for COX-2 expression in LPS-stimulated BV-2 microglia. This difference between the studies can be due to the time of stimulation, the compounds used and the cell types used, since we used primary rat microglia and the authors of this independent study used the BV-2 cell line. Although SB216763 has been shown to be selective for GSK-3 over many other kinases [13], there is still the possibility that other targets other than GSK-3 are involved in COX-2 regulation. Some studies have addressed the role of GSK-3 in COX-2 regulation, but no data have been published concerning the role of GSK-3 in mPGES-1 expression.

Besides GSK-3, another important target of Akt is mTOR. Interestingly, here we demonstrate that COX-2 and mPGES-1 immunoreactivity, as well as PGE_2 _and PGD_2_, were drastically increased by rapamycin, a selective inhibitor of mTOR, even at very low doses (Figure 3A-C). To our knowledge, this is the first demonstration that rapamycin increases the production of prostaglandins. It has been shown that mTOR inhibitors reduced NOS activity and iNOS expression induced by hypoxia and a mixture of cytokines in BV-2 cells and primary rat microglia, respectively [23,24]. However, Dello Russo et al. [23] did not find any increment in the COX-2 expression with the association of a mixture of cytokines and RAD001, another mTOR inhibitor. This discrepancy between the studies might be due to the type of stimulus and a difference in the chemical structure of the drugs used.

Although rapamycin enhanced COX-2 and mPGES-1 protein levels, NVP-BEZ235, the dual PI3K/mTOR inhibitor, strongly increased COX-2, but reduced mPGES-1 immunoreactivity induced by LPS in microglia (Figure 4A-B). In fact, we have already previously demonstrated that the regulation of these two enzymes is not strictly coupled [5]. Interestingly, PGE_2 _and PGD_2 _were enhanced in LPS-stimulated. One hypothesis to explain the enhancement of PGE_2 _with mPGES-1 reduction might be that the remaining mPGES-1 expression, together with cPGES and mPGES-2, which are expressed in microglia, would be enough to produce the high levels of PGE_2_. Moreover, the activity of the enzymes could also be increased by the compound.

There are other studies demonstrating that PGE_2 _could be enhanced even with mPGES-1 reduction. LY294002, a PI3K inhibitor, reduce mPGES-1 and enhance COX-2 expression, although the production of PGE_2 _and PGD_2 _is enhanced or not affected in LPS-stimulated microglia [5]. Moreover, it has been demonstrated that silencing mPGES-1 does not affect PGE_2 _production in IL-1β or TNFα-stimulated gingival fibroblasts [25]. Similarly, this study demonstrated that MK-886, an inhibitor of 5-lipoxygenase-activating protein, reduced mPGES-1 and increased COX-2 expression, but did not affect PGE_2 _synthesis. In fact, different data suggest that COX-2 might the rate-limiting enzyme in the synthesis of PGE_2 _[26-28].

Importantly, NVP-BEZ235 *per se *is able to increase PGE_2 _in non-stimulated cells. This result suggests that the dual PI3K/mTOR inhibitor might differently regulate the expression of inflammatory mediators in different conditions, since it reduces mPGES-1 induced by LPS and also upregulates this enzyme in non-stimulated conditions. That the increase in PGE_2 _mediated by NVP-BEZ235 is higher in non-stimulated in comparison to stimulated microglia is probably due to the fact that mPGES-1 is reduced in activated cells and increased in non-activated cells. Taken together, we provide evidence that the blockade of mTOR and/or PI3K/Akt enhances prostanoid production by microglia.

The results with rapamycin and NVP-BEZ235 represent important findings, since rapamycin is a commercially available drug used to prevent rejection of transplanted kidney and NVP-BEZ235 was recently identified [29] and is an orally bioavailable drug which is currently studied in clinical trials for advanced solid tumor patients. To date, only a few studies have demonstrated the effects of NVP-BEZ235 in non-tumor cells. Here we demonstrate that microglia may also be affected by a double PI3K/mTOR inhibition by modifying the production of inflammatory mediators in neuroinflammatory conditions. Considering that the arachidonic acid cascade products might have important roles in resolution and/or progression of neuroinflammation, the effects of these compounds should be investigated *in vivo*.

In conclusion, we provide novel evidence that Akt, GSK-3 and mTOR are important intracellular regulators of microglia activation. Our data provide significant information regarding the regulation of COX-2 and mPGES-1 via the Akt/mTOR and Akt/GSK-3 pathways, and suggest that interfering with these signalling cascades using pharmacological inhibitors could modulate the activation state of microglial cells during neuroinflammation.

## Abbreviations used

Akt: protein kinase B; COX: cyclooxygenase; cPGES: cytosolic prostaglandin E synthase; GSK-3: glycogen synthase kinase-3; iNOS: inducible nitric oxide synthase; IL: interleukin; LPS: lipopolysaccharide; mPGES: microsomal prostaglandin E synthase; mTOR: mammalian target of rapamycin PG: prostaglandin; PI3K: phosphatidylinositol 3-kinase; TNF: tumor necrosis factor.

## Competing interests

The authors declare that they have no competing interests.

## Authors' contributions

ACPdO, EC-J, and BLF participated in research design. The experiments were performed by ACPdO, LW, JL and HSB. Data were analysed by ACPdO, EC-J, JCMS and HSB. ACPdO, EC-J, JCMS, BLF, and KB wrote or contributed to the writing of the manuscript. In addition, ACPdO, KB, EC-J, JCMS, and BLF reviewed the data and discussed the manuscript. All authors have read and approved the final version of the manuscript.

## References

[B1] Liu B Hong JS Role of microglia in inflammation-mediated neurodegenerative diseases: mechanisms and strategies for therapeutic intervention J Pharmacol Exp Ther 2003 304 1 7 10.1124/jpet.102.035048 12490568

[B2] Laine J Kunstle G Obata T Noguchi M Differential regulation of Akt kinase isoforms by the members of the TCL1 oncogene family J Biol Chem 2002 277 3743 3751 10.1074/jbc.M107069200 11707444

[B3] Cantley LC The phosphoinositide 3-kinase pathway Science 2002 296 1655 1657 10.1126/science.296.5573.1655 12040186

[B4] Fukao T Koyasu S PI3K and negative regulation of TLR signaling Trends Immunol 2003 24 358 363 10.1016/S1471-4906(03)00139-X 12860525

[B5] de Oliveira AC Candelario-Jalil E Bhatia HS Lieb K Hull M Fiebich BL Regulation of prostaglandin E2 synthase expression in activated primary rat microglia: evidence for uncoupled regulation of mPGES-1 and COX-2 Glia 2008 56 844 855 10.1002/glia.20658 18383341

[B6] Seregi A Keller M Jackisch R Hertting G Comparison of the prostanoid synthesizing capacity in homogenates from primary neuronal and astroglial cell cultures Biochem Pharmacol 1984 33 3315 3318 10.1016/0006-2952(84)90099-6 6435636

[B7] Gebicke-Haerter PJ Bauer J Schobert A Northoff H Lipopolysaccharide-free conditions in primary astrocyte cultures allow growth and isolation of microglial cells J Neurosci 1989 9 183 194 2643682 10.1523/JNEUROSCI.09-01-00183.1989 PMC6569992

[B8] Livak KJ Schmittgen TD Analysis of relative gene expression data using real-time quantitative PCR and the 2(-Delta Delta C(T)) Method Methods 2001 25 402 408 10.1006/meth.2001.1262 11846609

[B9] Jope RS Yuskaitis CJ Beurel E Glycogen synthase kinase-3 (GSK3): inflammation, diseases, and therapeutics Neurochem Res 2007 32 577 595 10.1007/s11064-006-9128-5 16944320 PMC1970866

[B10] Chaudhry U Zhuang H Dore S Microsomal prostaglandin E synthase-2: cellular distribution and expression in Alzheimer's disease Exp Neurol 2010 223 359 365 10.1016/j.expneurol.2009.07.027 19664621 PMC2864315

[B11] Kubota K Kubota T Kamei D Murakami M Kudo I Aso T Morita I Change in prostaglandin E synthases (PGESs) in microsomal PGES-1 knockout mice in a preterm delivery model J Endocrinol 2005 187 339 345 10.1677/joe.1.06169 16423813

[B12] Zhang J Fujii S Wu Z Hashioka S Tanaka Y Shiratsuchi A Nakanishi Y Nakanishi H Involvement of COX-1 and up-regulated prostaglandin E synthases in phosphatidylserine liposome-induced prostaglandin E2 production by microglia J Neuroimmunol 2006 172 112 120 10.1016/j.jneuroim.2005.11.008 16371234

[B13] Coghlan MP Culbert AA Cross DA Corcoran SL Yates JW Pearce NJ Rausch OL Murphy GJ Carter PS Roxbee Cox L Selective small molecule inhibitors of glycogen synthase kinase-3 modulate glycogen metabolism and gene transcription Chem Biol 2000 7 793 803 10.1016/S1074-5521(00)00025-9 11033082

[B14] Ha SD Ng D Pelech SL Kim SO Critical role of the phosphatidylinositol 3-kinase/Akt/glycogen synthase kinase-3 signaling pathway in recovery from anthrax lethal toxin-induced cell cycle arrest and MEK cleavage in macrophages J Biol Chem 2007 282 36230 36239 10.1074/jbc.M707622200 17951252

[B15] Park SH Park-Min KH Chen J Hu X Ivashkiv LB Tumor necrosis factor induces GSK3 kinase-mediated cross-tolerance to endotoxin in macrophages Nat Immunol 2011 12 607 615 10.1038/ni.2043 21602809 PMC3258532

[B16] Ren F Duan Z Cheng Q Shen X Gao F Bai L Liu J Busuttil RW Kupiec-Weglinski JW Zhai Y Inhibition of glycogen synthase kinase 3 beta ameliorates liver ischemia reperfusion injury by way of an interleukin-10-mediated immune regulatory mechanism Hepatology 2011 54 687 696 10.1002/hep.24419 21567437 PMC3145016

[B17] Suh HS Choi S Khattar P Choi N Lee SC Histone deacetylase inhibitors suppress the expression of inflammatory and innate immune response genes in human microglia and astrocytes J Neuroimmune Pharmacol 2010 5 521 532 10.1007/s11481-010-9192-0 20157787 PMC3115474

[B18] Yuskaitis CJ Jope RS Glycogen synthase kinase-3 regulates microglial migration, inflammation, and inflammation-induced neurotoxicity Cell Signal 2009 21 264 273 10.1016/j.cellsig.2008.10.014 19007880 PMC2630396

[B19] Takada Y Fang X Jamaluddin MS Boyd DD Aggarwal BB Genetic deletion of glycogen synthase kinase-3beta abrogates activation of IkappaBalpha kinase, JNK, Akt, and p44/p42 MAPK but potentiates apoptosis induced by tumor necrosis factor J Biol Chem 2004 279 39541 39554 10.1074/jbc.M403449200 15252041

[B20] Rao R Hao CM Breyer MD Hypertonic stress activates glycogen synthase kinase 3beta-mediated apoptosis of renal medullary interstitial cells, suppressing an NFkappaB-driven cyclooxygenase-2-dependent survival pathway J Biol Chem 2004 279 3949 3955 14607840 10.1074/jbc.M309325200

[B21] Tang Q Gonzales M Inoue H Bowden GT Roles of Akt and glycogen synthase kinase 3beta in the ultraviolet B induction of cyclooxygenase-2 transcription in human keratinocytes Cancer Res 2001 61 4329 4332 11389054

[B22] Thiel A Heinonen M Rintahaka J Hallikainen T Hemmes A Dixon DA Haglund C Ristimaki A Expression of cyclooxygenase-2 is regulated by glycogen synthase kinase-3beta in gastric cancer cells J Biol Chem 2006 281 4564 4569 16371352 10.1074/jbc.M512722200

[B23] Dello Russo C Lisi L Tringali G Navarra P Involvement of mTOR kinase in cytokine-dependent microglial activation and cell proliferation Biochem Pharmacol 2009 78 1242 1251 10.1016/j.bcp.2009.06.097 19576187

[B24] Lu DY Liou HC Tang CH Fu WM Hypoxia-induced iNOS expression in microglia is regulated by the PI3-kinase/Akt/mTOR signaling pathway and activation of hypoxia inducible factor-1alpha Biochem Pharmacol 2006 72 992 1000 10.1016/j.bcp.2006.06.038 16919605

[B25] Bage T Modeer T Kawakami T Quezada HC Yucel-Lindberg T Regulation of prostaglandin E synthases: effects of siRNA-mediated inhibition of microsomal prostaglandin E synthase-1 Biochim Biophys Acta 2007 1773 1589 1598 10.1016/j.bbamcr.2007.07.008 17707523

[B26] Degousee N Martindale J Stefanski E Cieslak M Lindsay TF Fish JE Marsden PA Thuerauf DJ Glembotski CC Rubin BB MAP kinase kinase 6-p38 MAP kinase signaling cascade regulates cyclooxygenase-2 expression in cardiac myocytes in vitro and in vivo Circ Res 2003 92 757 764 10.1161/01.RES.0000067929.01404.03 12649265

[B27] Mancini A Jovanovic DV He QW Di Battista JA Site-specific proteolysis of cyclooxygenase-2: a putative step in inflammatory prostaglandin E(2) biosynthesis J Cell Biochem 2007 101 425 441 10.1002/jcb.21191 17177291

[B28] Sevigny MB Li CF Alas M Hughes-Fulford M Glycosylation regulates turnover of cyclooxygenase-2 FEBS Lett 2006 580 6533 6536 10.1016/j.febslet.2006.10.073 17113084

[B29] Maira SM Stauffer F Brueggen J Furet P Schnell C Fritsch C Brachmann S Chene P De Pover A Schoemaker K Identification and characterization of NVP-BEZ235, a new orally available dual phosphatidylinositol 3-kinase/mammalian target of rapamycin inhibitor with potent in vivo antitumor activity Mol Cancer Ther 2008 7 1851 1863 10.1158/1535-7163.MCT-08-0017 18606717

